# Impact of Protein Domains on PE_PGRS30 Polar Localization in Mycobacteria

**DOI:** 10.1371/journal.pone.0112482

**Published:** 2014-11-12

**Authors:** Flavio De Maio, Giuseppe Maulucci, Mariachiara Minerva, Saber Anoosheh, Ivana Palucci, Raffaella Iantomasi, Valentina Palmieri, Serena Camassa, Michela Sali, Maurizio Sanguinetti, Wilbert Bitter, Riccardo Manganelli, Marco De Spirito, Giovanni Delogu

**Affiliations:** 1 Institute of Microbiology, Universita' Cattolica del Sacro Cuore, Rome, Italy; 2 Institute of Physics, Universita' Cattolica del Sacro Cuore, Rome, Italy; 3 Department of Medical Microbiology and Infection Control, VU University Medical Center, Amsterdam, Netherlands; 4 Department of Molecular Medicine, University of Padua, Padua, Italy; Public Health England, United Kingdom

## Abstract

PE_PGRS proteins are unique to the *Mycobacterium tuberculosis* complex and a number of other pathogenic mycobacteria. PE_PGRS30, which is required for the full virulence of *M. tuberculosis* (*Mtb*), has three main domains, *i.e.* an N-terminal PE domain, repetitive PGRS domain and the unique C-terminal domain. To investigate the role of these domains, we expressed a GFP-tagged PE_PGRS30 protein and a series of its functional deletion mutants in different mycobacterial species (*Mtb, Mycobacterium bovis* BCG and *Mycobacterium smegmatis*) and analysed protein localization by confocal microscopy. We show that PE_PGRS30 localizes at the mycobacterial cell poles in *Mtb* and *M. bovis* BCG but not in *M. smegmatis* and that the PGRS domain of the protein strongly contributes to protein cellular localization in *Mtb*. Immunofluorescence studies further showed that the unique C-terminal domain of PE_PGRS30 is not available on the surface, except when the PGRS domain is missing. Immunoblot demonstrated that the PGRS domain is required to maintain the protein strongly associated with the non-soluble cellular fraction. These results suggest that the repetitive GGA-GGN repeats of the PGRS domain contain specific sequences that contribute to protein cellular localization and that polar localization might be a key step in the PE_PGRS30-dependent virulence mechanism.

## Introduction

Despite many research and sanitary efforts, tuberculosis (TB) remains one of the deadliest human infectious diseases far from being defeated [1]. The poor knowledge of the biology of its causative agent, *Mycobacterium tuberculosis* (*Mtb*), is a main obstacle toward the development of improved control strategies [2], [3]. In this context, a better understanding of surface exposed, secreted and cell wall associated proteins is classically a key step to dissect the mechanisms of pathogenesis of bacteria and to identify antigens that may serve as candidate vaccines [4], [5]. The complexity of the mycobacterial cell wall is such that only recently it has been possible to solve its structure [6], [7], including a peculiar outer membrane referred to as mycomembrane. Consequently, we still have limited knowledge regarding the proteins and protein apparatuses localizing in the mycomembrane and the molecular determinants mediating host-pathogen interactions [8]. The recent discovery of the ESX secretion systems is shedding light on the mechanism whereby *Mtb* translocate effector proteins that are secreted or exposed on its surface and that can interfere with host components [9]–[12]. The results of these studies are leading to the development of new vaccines and drug targets [13], [14], emphasizing the impact that this line of research may have in the control of TB.

Among the cell wall associated proteins are the PE_PGRSs, a family of around 60 proteins found only in members of the *Mtb* comple*x*, in *Mycobacterium ulcerans* and *Mycobacterium marinum* [15]–[18]. PE_PGRSs are characterized by a highly conserved PE domain, a central polymorphic PGRS domain and a unique C-terminal domain that may vary in size from few to up to 300 amino acids [17]. Studies carried out with PE_PGRS33 showed that the PE domain is required for the correct protein localization in the mycobacterial cell wall [19]–[21], although only the PGRS domain appears to be properly exposed for interaction with host components [22]. Indeed, PE_PGRS33 shows immunomodulatory properties thanks to its ability to interact with TLR2, which may trigger macrophage cell death [16], [23]–[26].

Among the few PE_PGRSs for which experimental evidences are available, PE_PGRS30 is required for the full virulence of *Mtb* [27]. PE_PGRS30, encoded by the gene Rv1651c in *Mtb* H37Rv, is a protein of 1011 amino acids composed by a PE domain (90 aa), followed by a domain of 39 amino acids containing the highly conserved GRPLI motif (TM, trans-membrane domain) that is probably involved in the anchorage of the protein to the mycobacterial cell wall [17], [27], [28]. The central region of the protein is formed by the PGRS domain (566 aa), which is followed by a large unique C-terminal domain (316 aa). While we await a functional characterization of the different protein domains, it was with surprise that the large unique C-terminal domain was found dispensable for the PE_PGRS30-dependent virulence phenotype [27].

The role and precise localization of PE_PGRS proteins is still elusive as well as the role of their different domains in this process. Objective of this study is the characterization of the domains involved in the cellular localization of PE_PGRS30.

## Materials and Methods

### Construction of plasmids expressing PE_PGRS30 and its chimeras fused with green fluorescent protein (GFP)

The PE_PGRS30 full length gene and selected fragments of its open reading frame were amplified from the *Mtb* H37Rv genomic DNA [15] using primers indicated in table S1 and cloned using standard procedures. Briefly, the forward primer was designed to anneal to the upstream region of the Rv1651c so to amplify its putative promoter sequence and contained the *Hind*III restriction site adaptor sequence. Reverse primers were designed to anneal to different positions of the Rv1651c coding sequence and contained the *Xba*I adaptor sequence. PCR products were amplified using Vent polymerase (New England Biolab, Beverly, MA) and cloned in pCR blunt vector (Life technologies). The GFP gene was amplified from the pJWtPAGFP vector [29] using primers containing the *Nhe*I and *Bam*HI restriction sites at 5′ and 3′, respectively. The PE_PGRS30-derived fragments were inserted in the pMV206 medium copy episomal plasmid [30] in frame and upstream of the GFP coding sequence. The gene cassettes, containing the Rv1651c putative promoter sequence and the PE_PGRS30-derived fragments, were also inserted in the integrative plasmid pMV361 downstream of the *hsp*60 promoter sequence and upstream and in frame with the sequence coding the haemagglutinin (HA) epitope.

### Bacterial strains

The constructed recombinant plasmids were used to transform *Mtb* H37Rv, *Mycobacterium bovis* BCG Pasteur and *Mycobacterium smegmatis* mc^2^155 using standard procedures [19]. Transformants were selected on 7H11 agar media supplemented with 10% OADC (Microbiol, Cagliari, Italy) containing 50 µg/ml hygromycin B (Sigma–Aldrich, Saint Louis, MO). Single individual antibiotic-resistant colonies were isolated and subcultured in a 7H9 media supplemented with 10% ADC (Microbiol, Cagliari, Italy) and 0,05% Tween 80 containing hygromycin B (50 µg/ml) and incubated at 37°C. Mycobacteria cultures were stocked at −80°C in 20% glycerol.

### Construction of the DNA plasmid vector encoding the PE_PGRS30 C-terminal unique domain (ptPA-30^Cter^)

A plasmid DNA coding the C-terminal domain of PE_PGRS30 was constructed following standard procedures. Briefly, a 918 bp fragment corresponding to the coding sequence of the C-terminus 306 amino acids domain of PE_PGRS30 was amplified using the primers indicated in table S1. The DNA fragment was amplified from the *Mtb* H37Rv genomic DNA using Vent polymerase and cloned in pCRblunt (Life Technologies). The DNA fragment was then cleaved with *Nhe*I and *Bam*HI and cloned in pJW4303 to obtain ptPA^30Cter^ [29]. Endotoxin-free plasmid DNA was prepared and purified with the Qiagen EndoFree Plasmid Maxi Kit (Qiagen, Chatsworth, US) for the ptPA^30Cter^ and ptPA-GFP constructs [29].

### Immunization of mice

Specified pathogen-free female BALB/c mice were obtained from Enclosure Labs of the Università Cattolica del Sacro Cuore, Rome and immunized at the age of eight weeks. The animals were housed in a temperature-controlled environment with 12 h light/dark cycles, and received food and water ad libitum. All animal experiments were authorized by the Ethical Committee of the Università Cattolica del Sacro Cuore and performed in compliance with the legislative decree of the Italian Government 27 January 1992, n. 116 and the Health Minister memorandum 14 May 2001, n. 6. All manipulations were performed under isoflurane anesthesia, and all efforts were made to minimize suffering. Three Balb/c mice per group (pTPA-GFP and ptPA^30Cter^, total six mice) were immunized by three intramuscular injection of 100 µg of plasmid DNA and bled 4–10 weeks following the third immunization by the tail vein to collect sera, as previously indicated [31].

### Immunoblotting

The *Mtb* recombinant strains were cultured in 7H9 media containing Tween80 (without ADC) until mid-log phase and cells harvested by centrifugation [32]. To obtain whole cell lysates, cell pellets were washed with PBS and directly re-suspended in Laemmli buffer and boiled for 10 minutes. To obtain the cytosolic fraction, cell pellets were resuspended in lysis buffer (10 mM Tris-HCl, 5 mM EDTA, protease inhibitors cocktail, pH 9.5) containing 0,1 mm Silica/Zirconia beads (Biospec products) and subjected to three cycles of homogenization with the Mini-Beadbeater (Biospec products). After centrifugation to remove the insoluble fraction supernatants were filtered through 0.22 µm filters (Cellulose acetate membrane sterile syringe filter, VWR International). Proteins samples were separated by SDS-PAGE and transferred to nitrocellulose membranes by western blot. Membranes were probed with polyclonal sera (1∶200) obtained from mice immunized with ptPA^30Cter^ or ptPA-GFP and then anti-mouse IgG-Peroxidase (Sigma–Aldrich, Saint Louis, MO) was used as a secondary antibody. Immunoblot developed using Supersignal West Dura Extended Duration Substrate (Thermo scientific). Membranes were probed with polyclonal sera (1∶200) obtained from mice immunized with ptPA^30Cter^ or monoclonal anti-HA epitope antibody (Covance).

### Immunofluorescence

Recombinant *Mtb* strains, expressing PE_PGRS30 and its chimera _30_PE_Ct tagged with HA epitope, were plated in chamber slides as indicated above and then fixed with 4% paraformaldehyde and washed with PBS. After blocking with BSA 0.3%, plates were incubated with anti-HA epitope (1∶200) (Covance). After washing with PBS, slides were probed with the secondary antibody Alexa Fluor 546 donkey anti-mouse (Life technologies) and then Prolong gold anti-fade reagent (Life technologies) was added before closing the slides. Chamber slides were observed with a confocal microscope.

### Cells cultures and mycobacteria infection

J774 cells (ATCC) were grown in RPMI-1640 medium (Euroclone Milan, Italy) supplemented with 10% fetal calf serum (FCS), glutamine (2 mM), and sodium pyruvate (1 mM) (Euroclone Milan, Italy) and kept in a humidified atmosphere containing 5% CO_2_ at 37°C [27]. Cells were plated in chamber slides (1,2×10^6^ cell/ml) in media without antibiotics and then infected with the recombinant *Mtb* strains expressing the GFP-tagged proteins at a multiplicity of infection (MOI) of 5∶1. After 1 hour of incubation, cells were washed with warm PBS and after adding fresh media (RPMI 2% FCS without antibiotics) plates were incubated at 37°C (5% CO2). At different time points, cells were washed with PBS, fixed with 4% paraformaldehyde and chamber slides closed and observed at the confocal microscope as indicated above.

### Confocal microscopy and image analysis

The recombinant mycobacterial strains (*M. tuberculosis* H37Rv, *M. bovis* BCG and *M. smegmatis* mc^155^) expressing PE_PGRS30 and its chimeras fused with GFP, were grown in 7H9/ADC/Tween80 and at mid-log phase these strains were plated on chamber slides pre-treated with polylisine (Sigma–Aldrich, Saint Louis, MO). Subsequently chamber slides were incubated for 24 hours at 37°C, then fixed with 4% parafolmaldehyde and washed with phosphate buffered saline (PBS). Chamber slides were closed and observed with a confocal microscope.

Images were collected by using an inverted confocal microscope (DMIRE2, Leica Microsystems, Wetzlar, Germany) equipped with a 40× oil immersion objective (NA 1.25). For GFP excitation a He/Ne laser at 476 nm was used. Internal photon multiplier tubes collected 8-bit unsigned images at 400 Hz scan speed in an emission range comprised between 500 nm and 550 nm. Imaging was performed at room temperature. Image processing was performed with ImageJ software; image background values (defined as intensities below 7% of the maximum intensity) were set to zero and colored in black [33]. Intensity profiles were measured on bacteria entire length with ‘line profile’ tool [34]. To obtain a representative index of protein polar localization, a ratio R was calculated as follows: R = I_pole_/I_cyto_ where I_pole is the GFP emission intensity at bacterium pole, considered as the value in correspondence of the border of the bacterium and I_cyto_ is the GFP emission intensity in the middle part of the bacterium (measured at the 50% of the bacterium length). At least 20 line profiles were analyzed for each construct. R values are reported for each line profile and their mean ± standard deviation (SD) were determined and utilized for two-tailed Student's *t*-test analysis (GraphPad).

Localization of the anti-mouse AlexaFluor-546-tagged antibodies (Life technologies) outside the mycobacterial cell was assessed by acquiring images with a 63× oil immersion objective (NA 1.4) using an excitation wavelength of 543 nm (Ar/ArKr laser) and with photomultiplier emission range comprised between 560 and 700 nm. Image background was corrected as defined above and fluorophore localization was evaluated with ImageJ software using overlap between red channel and transmission images.

## Results

### Expression of PE_PGRS30-derived GFP chimeras in mycobacterial strains

**Figure 1:**
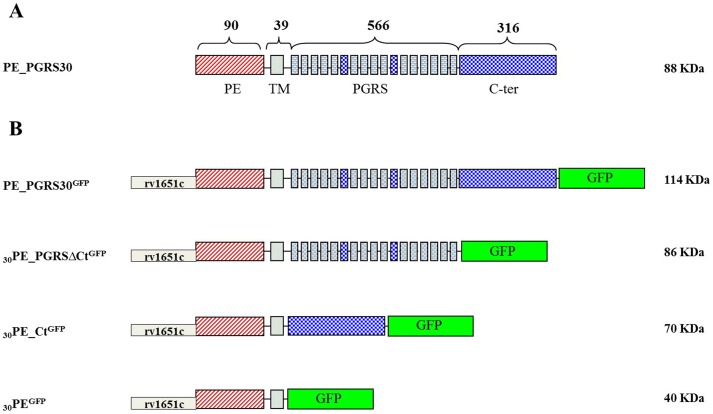
Scheme showing the constructs expressing PE_PGRS30 used in this study. Schematic representation of native full length PE_PGRS30 gene with the indication of the different protein domains (A). List of constructs generated in pMV206 and expressing the PE_PGRS30 functional chimeras, tagged with green fluorescent protein (B). The constructs were transformed in *Mtb* H37Rv, *M. smegmatis* and *M. bovis* BCG.

To determine PE_PGRS30 localization in the mycobacterial cell and the impact of the different protein domains on this process, the PE_PGRS30 native gene, and its functional deletion mutants [27], were fused to the GFP coding sequence in the medium copy number plasmid pMV206 (figure 1) [30]. These plasmids were electroporated in *Mtb* H37Rv, *M. bovis* BCG and *M. smegmatis* mc^2^155 to obtain strains expressing PE_PGRS30-derived GFP-tagged proteins. Expression of these chimeras was under the control of the PE_PGRS30 (Rv1651c) putative promoter [27]. Correct protein expression was demonstrated by immunoblot using an anti-GFP antibody on whole cell lysates of recombinant *Mtb*, *M. smegmatis* and BCG strains (Figure S1).

### PE_PGRS30 localize at cellular poles in *Mtb* and in *M. bovis* BCG but not in *M. smegmatis*

**Figure 2:**
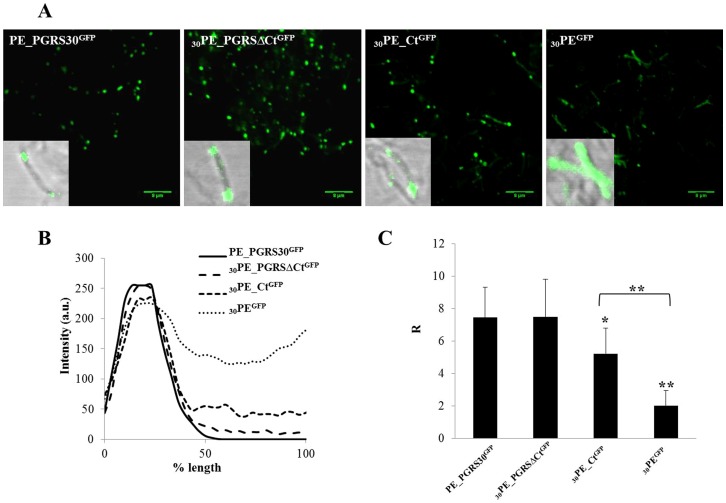
Polar localization of PE_PGRS30^GFP^ spatial distribution in *Mtb*. A) Confocal images of *Mtb* H37Rv expressing PE_PGRS30^GFP^ and its functional GFP-tagged chimeras obtained with a 63× objective. In the inbox, a 100× image obtained overlapping green channel and transmission image is shown. B) Sample line profile obtained quantifying the fluorescence along mycobacterial cell. C) Ratio between the GFP emission intensity at bacterium pole (considered as the value 200 nm far from the bacterium border) and GFP emission intensity in the cytoplasm (measured at the 50% of the bacterium length). Twenty R values were analyzed for each *Mtb* strain under study. Two-tailed Student's *t*-test was used to analyze R ratio (* *p*<0.05, ** *p<*0.01).

The recombinant *Mtb* strains expressing PE_PGRS30 and its chimeras fused with GFP were grown until mid-log phase and plated in chamber slides and then observed at fluorescence confocal microscope. As shown in figure 2A, polarization of the GFP signal was observed for the strain expressing PE_PGRS30^GFP^ and a similar pattern was observed for *Mtb-*_30_PE_PGRSΔCT^GFP^ and, to a lesser extent, for *Mtb-*_30_PE_CT^GFP^. Conversely, a fluorescence diffused throughout the mycobacterial cell was observed for the *Mtb-*_30_PE^GFP^. Analysis of the fluorescence pattern was performed using line profile software (ImageJ software), in order to evaluate the polar localization of the protein spatial distribution. As shown in the line profile (figure 2B), comparison of the fluorescence pattern indicated a less pronounced polarization in the *Mtb-*_30_PE^GFP^ strain compared with the full length PE_PGRS30 chimera and the other two chimeras. R values (mean ± SD) are reported in figure 2C for each sample and in table S2, with higher R values indicating more pronounced polar localization of the GFP-tagged protein. A significant difference is observed only for *Mtb-*_30_PE^GFP^ and *Mtb-*_30_PE_CT^GFP^.

As shown in figure S2, the BCG recombinant strains expressing PE_PGRS30 and its chimeras fused with GFP were analyzed as for *Mtb* and the results obtained indicate a similar pattern of protein localization to that observed in *Mtb*, though a less pronounced polar localization was observed for the _30_PE_CT^GFP^ chimera.

**Figure 3:**
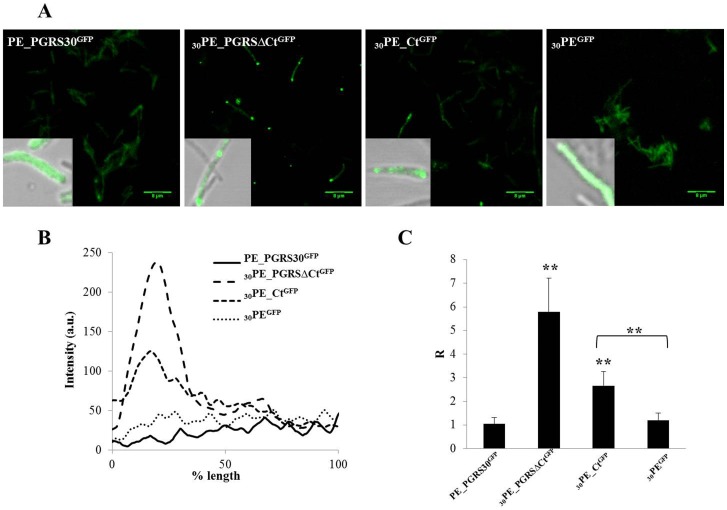
Polar localization of PE_PGRS30^GFP^ spatial distribution in *M. smegmatis*. A) Confocal images of *M. smegmatis* expressing PE_PGRS30^GFP^ and its functional GFP-tagged chimeras obtained with a 63× objective. In the inbox, a 100× image obtained overlapping green channel and transmission image is shown. B) Sample line profile obtained quantifying the fluorescence along mycobacterial cell. C) Ratio between the GFP emission intensity at bacterium pole (considered as the value 200 nm far from the bacterium border) and GFP emission intensity in the cytoplasm (measured at the 50% of the bacterium length). Twenty R values were analyzed for each *M. smegmatis* strain under study. Two-tailed Student's *t*-test was used to analyze R ratio (* *p*<0.05, ** *p<*0.01).

Figure 3A shows the results obtained with the *M. smegmatis* recombinant strains expressing the chimeras under study. Interestingly, expression and localization profiles in this species were radically different; expression of the full length protein (PE_PGRS30^GFP^), of _30_PE_CT^GFP^ and _30_PE^GFP^ resulted in a diffused fluorescence with little or no polarization (figure 3B).

Taken together these results indicate that expression of the PE_PGRS30 chimeras in *Mtb* and BCG follows a similar pattern, which is different to what observed in *M. smegmatis*, and that the PE domain does not contain the information sufficient to warrant polar localization of the full PE_PGRS30 protein.

### The PGRS domain contributes to PE_PGRS30 cellular localization in *Mtb*

**Figure 4:**
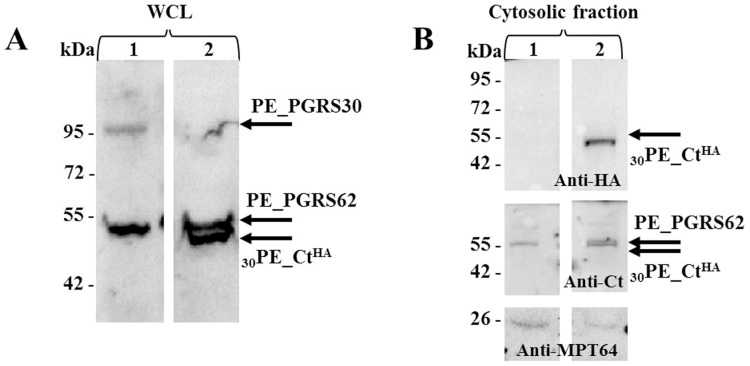
Immunoblot showing expression of PE_PGRS30 by *Mtb*. A) Immunoblot analysis on whole cell lysates of *Mtb* expressing PE_PGRS30 and its HA-tagged chimeras with anti-C-terminal domain primary antibody. B) Immunoblot analysis on cytoplasmatic fraction of *Mtb* expressing PE_PGRS30 and its HA_tagged chimeras probed with anti-HA antibody, anti-C-terminal domain and anti-MPT64 sera; 1: *Mtb*-PE_PGRS30^HA^; 2: *Mtb*-_30_PE_CT^HA^; Arrows indicate the band corresponding to PE_PGRS30^HA^ and PE_CT^HA^ and PE_PGRS62.

Compared with most PE_PGRS proteins, PE_PGRS30 contains, downstream of the PGRS domain, a large unique C-terminal domain (306 aa) of unknown function, and whose role in mediating protein localization has not been investigated. A plasmid DNA expressing only the PE_PGRS30 C-terminal domain (figure S3A) was used to immunize mice following standard procedures [29] and the antiserum raised was used to probe in immunoblots whole cell lysates of *Mtb* containing the different PE_PGRS30 constructs (figure 4A). A band of ≈90 kDa was detected in the whole lysate of *Mtb* expressing PE_PGRS30, that was not detected in any other of the *Mtb* strains tested (figure 4A), indicating that PE_PGRS30 cannot be detected in immunoblots with this polyclonal sera unless the protein is overexpressed as in *Mtb-*PE_PGRS30, where overexpression is warranted by the presence, upstream of the gene cassette inserted in pMV361, of the *hsp60* promoter. Interestingly, in all the *Mtb* strains a much stronger signal at 52 kDa was detected, probably corresponding to the gene product of Rv3812, which is a PE-unique protein containing a C-terminal domain highly homologous to that of PE_PGRS30 [35], [36]. The whole cell lysate of *Mtb*-_30_PE_CT, expressing the functional deletion mutant under similar conditions as the full length protein (pMV361), showed a band of ≈50 kDa, theoretically corresponding to the expected size of the _30_PE_CT chimera, and with a signal intensity similar to that observed for the Rv3812 gene product (figure S3B). Immunoblot analysis carried out on the soluble fraction of the *Mtb* lysates (figure 4B), showed the presence of the PE_CT chimera with the anti-HA and anti-Ct sera, though the full length protein and the other chimeras could not be detected. Again, the anti-Ct sera identified a band at ≈50 kDa as in figure 4A. These results suggest that the full length protein remains mostly associated with cellular debris compared with the _30_PE_CT chimera.

### The C-terminal domain of PE_PGRS30 is not exposed on the mycobacterial surface

**Figure 5:**
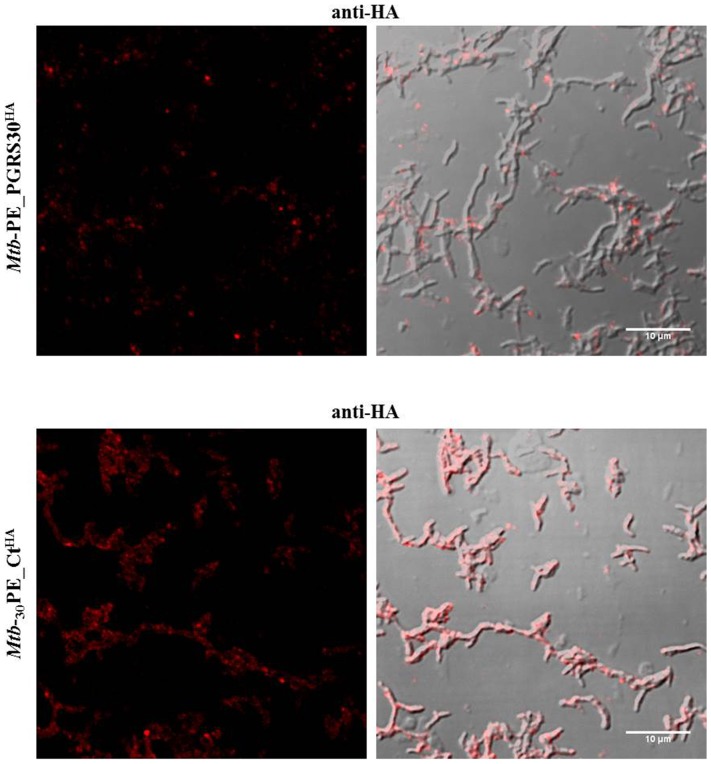
Immunofluorescence using anti-HA antibodies. *Mtb*-PE_PGRS30^HA^ (A) and *Mtb*-_30_PE_CT^HA^ (B) were subjected to immunofluorescence using anti-HA antibodies. Confocal images were acquired with a 63× oil immersion objective and localization was evaluated with ImageJ software using overlap between red channel (left panel) and transmission images.

To assess whether the large unique C-terminal domain was available on the mycobacterial surface, the recombinant *Mtb* expressing PE_PGRS30 and *Mtb* expressing PE_CT fused with the HA epitope were assayed in immunofluorescence studies using the anti-HA antibody. As shown in figure 5A, analysis at the fluorescence confocal microscope indicated that no significant signal was detected on the surface of *Mtb* expressing full length PE_PGRS30 using an anti-HA primary antibody. Conversely, *Mtb* expressing _30_PE_CT^HA^ chimeras showed a fluorescence along the outside mycobacterial cell wall (figure 5B). These results suggest that the C-terminal domain of PE_PGRS30 is not available on the mycobacterial surface, unless the PGRS domain is lacking as in *Mtb*_30_PE_CT^HA^, further establishing the key role of the PGRS domain for the correct localization of PE_PGRS30.

### PE_PGRS30 and PE_PGRS33 have different localization in *Mtb*

**Figure 6:**
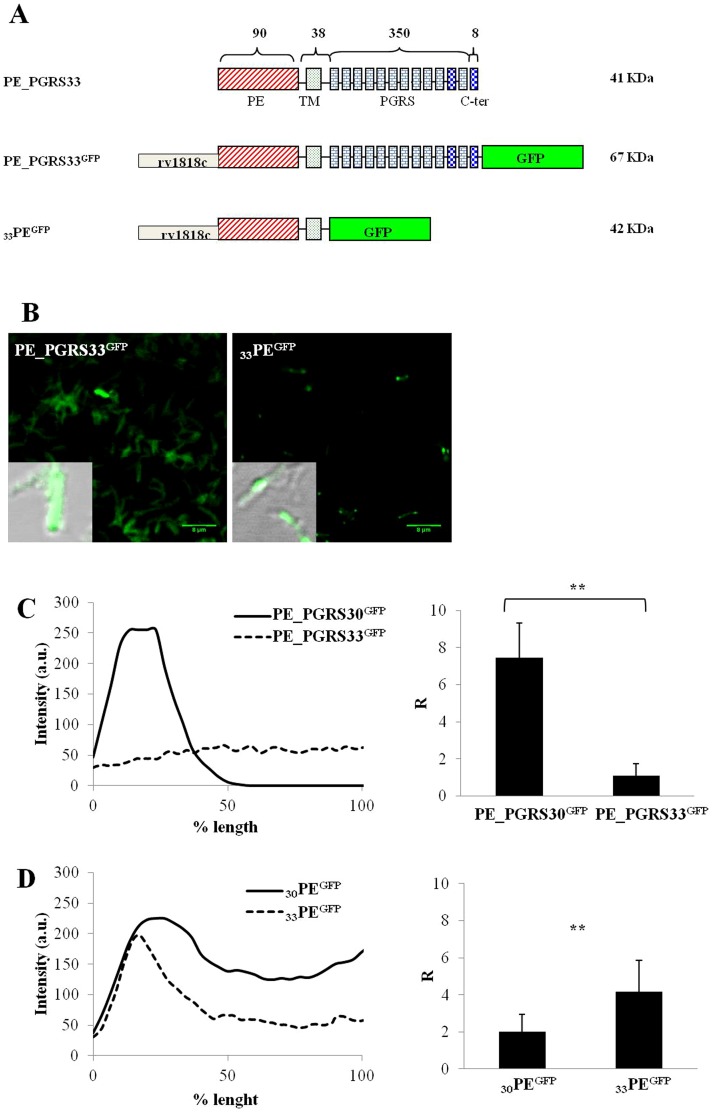
Differential polar localization between PE_PGRS30 and PE_PGRS33 spatial distribution in *Mtb*. A) Schematic representation of the PE_PGRS33-derived chimeras expressed in *Mtb* H37Rv. B) Confocal images of *Mtb* H37Rv expressing PE_PGRS33^GFP^ and _33_PE^GFP^ using a 63× objective. In the inbox, a 100× image obtained overlapping green channel and transmission image is shown. Sample line profile and ratio between the GFP emission intensity at the bacterium pole and the GFP emission intensity in the cytoplasm between PE_PGRS30^GFP^ and PE_PGRS33^GFP^ (C) and between _30_PE^GFP^ and _33_PE^GFP^ (D). Twenty R values were analyzed for each *Mtb* strain under study. Two-tailed Student's *t*-test was used to analyze R ratio (* *p*<0.05, ** *p<*0.01).

Previous studies demonstrated that the PE domain of PE_PGRS33 contains the information sufficient to drive localization of the protein to the mycobacterial cell wall [19]–[21] and that it is possible to use this domain to deliver protein or protein domains to the mycobacterial surface [37]. To investigate whether the pattern of protein polarization observed for PE_PGRS30 was similar to that observed for another well studied PE_PGRS protein (PE_PGRS33), we generated a plasmid expressing the PE_PGRS33^GFP^ chimera under the control of its physiological promoter (p*_rv1818c_*) (figure 6A, B). This construct is different from that used in previous studies where PE_PGRS33^GFP^ was overexpressed under the control of the *hsp60* promoter [19]. The *Mtb* strain transformed with this plasmid was analyzed by confocal microscopy and protein polarization measured with the line profile software (figure 6C). Surprisingly, differently from what observed with *Mtb*-PE_PGRS30^GFP^, no polarization was observed for the *Mtb*-PE_PGRS33^GFP^, where fluorescence was found diffused throughout the cell. To assess whether the differential polarization was due to the PE domain, an *Mtb* strain expressing the first 140 amino acids of PE_PGRS33 fused to GFP, under the control of the Rv1818c promoter, was generated and analyzed at confocal fluorescence microscopy (figure 6D). The _33_PE^GFP^ showed a strong polarization (figure 6B), that was not observed for _30_PE^GFP^ (figure 1A), clearly indicating that under physiological conditions the PGRS domain of PE_PGRS33 contributes to protein localization on the mycobacterial cell wall. The different degree of polarization observed for _30_PE^GFP^ and _33_PE^GFP^ suggests that amino acids differences in the PE domain may also impact protein localization (figure S4).

### PE_PGRS30 and PE_PGRS33 have a different polar localization during infection

**Figure 7:**
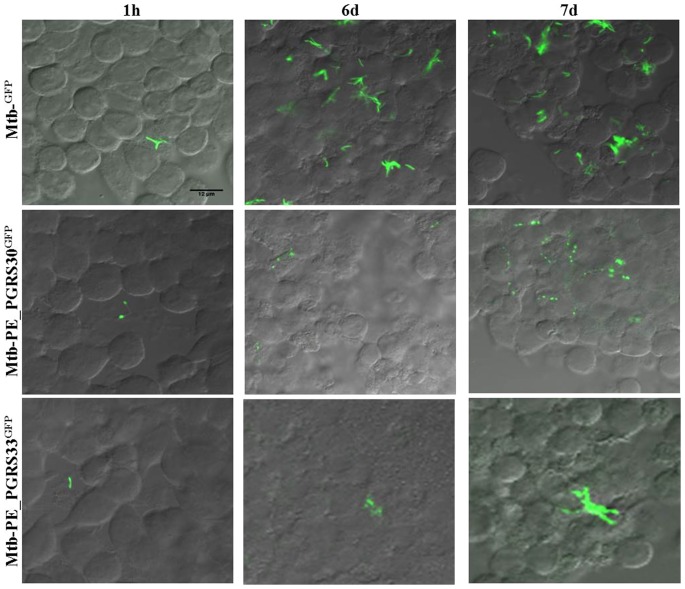
Polar localization of PE_PGRS30 during *Mtb* macrophage infection. Macrophages (J774) were infected with the *Mtb* ^GFP^, *Mtb*-PEGRS30^GFP^ and *Mtb*-PE_PGRS33^GFP^ and cells washed and fixed at 1 h and 6 days post-infection. Supernatants from infected macrophages at 6 days post-infection were harvested and used to infect fresh J774 macrophages, that 1 day later were washed and harvested. Slides containing infected macrophages harvested at the different time-points were analyzed at the confocal microscopy and images were obtained using a 63× objective.

Protein localization in mycobacteria may be affected by expression level or by interaction with other protein partners or cellular components, which in turn depend on the environment [38]. To assess protein localization in conditions mimicking the intracellular environment typically encountered by bacteria, *Mtb* expressing PE_PGRS30^GFP^ and PE_PGRS33^GFP^ were used to infect the murine macrophages cell line J774. An *Mtb* strain expressing GFP only was used as a control. Infected cells were fixed at different time points (1 hour and 6 days post-infection, and day 1 post-reinfection) and then observed at the confocal microscope as previously described. The first two time points mimic the early (1 hour) and the late (6 days) phase of infection, respectively. As expected, infection with the *Mtb* ^GFP^ control strain resulted in bacteria showing diffused and homogeneous fluorescence throughout the bacilli, and no changes in fluorescence diffusion were observed between 1 hour and 6 days post-infection (figure 7). A clear polarization was observed in *Mtb*-PE_PGRS30^GFP^ infecting macrophages at the early time point, and the polar localization was found more pronounced at 6 days post-infection. Conversely, diffused fluorescence was observed at 1 hour and 6 days post-infection for the *Mtb*-PE_PGRS33^GFP^ strain (figure 7). Since bacteria used to infect cells were obtained from glycerol stocks prepared from cultures grown in axenic media, it cannot be excluded that protein localization observed at 1 hour post-infection may represent the *Mtb* status in axenic media rather than a physiological situations. Hence, we collected the supernatants of the macrophage-infected culture at 6 days post-infection, that contain many bacteria released by dying macrophages, and used it to infect fresh macrophages. One day after infection, macrophages were fixed and analyzed at the confocal microscope. Polarization of PE_PGRS30^GFP^ was found even more pronounced under this condition, while *Mtb*-PE_PGRS33^GFP^ showed a diffused and homogenous fluorescence throughout the cell.

## Discussion

Since their identification in the *Mtb* genome, PE_PGRS proteins have been implicated in the mechanism of pathogenesis of TB and included in an hypothetical panel of surface mycobacterial antigens involved in immune evasion strategies [15]–[17]. In this study, using a panel of GFP-tagged proteins, we investigate the localization of PE_PGRS30 in three mycobacterial species (*Mtb*, *M. bovis* BCG and *M. smegmatis*) and analyzed the impact of the different protein domains on protein polarization on the bacterial cells. We show that both the PGRS and C-terminal unique domain of PE_PGRS30 contribute to protein localization and that the C-terminal domain is not available on the mycobacterial surface. Moreover, using GFP-protein chimeras we demonstrate that PE_PGRS30 localize at the bacterial poles during infection in macrophages, while PE_PGRS33 remains homogeneously distributed on the mycobacterial surface. These results provide further insights on PE_PGRS protein localization and suggest the functional diversity between PE_PGRS proteins.

Recent data obtained using an *Mtb* mutant strain demonstrated that PE_PGRS30 is required for the full virulence of *Mtb* and for intracellular survival of the bacilli in macrophages [27]. While the exact mechanism whereby PE_PGRS30 exerts its activity remains to be elucidated, some results obtained in *M. marinum* suggest that PE_PGRS proteins may be secreted as effector molecules through the ESX5 apparatus, a type seven secretion system (T7SS) [9], [39]. These results are in line with the discovery that in MTB complex, *M. marinum* and *M. ulcerans*, PE_PGRS (and PPE_MPTR) proteins emerged and coevolved in parallel with the ESX5 secretion system [40], [41], suggesting a functional link between the most recent T7SS (ESX5) and these two protein subfamilies [10]. Indeed, ESX-5 was elegantly shown to be required for the export of several immunogenic PE and PPE proteins and for the full virulence of *Mtb* [42], [43]. In this context, PE_PGRSs would be a substrate for ESX5 which mediates protein secretion or translocation to the surface, as it has been shown in *M. marinum* [9], [44]. However, ESX5-dependent PE_PGRS secretion in *Mtb* is still debated, with some experimental data supporting the ESX5-dependent secretion of PE_PGRSs [45] and other suggesting that inactivation of ESX5 in *Mtb* has no obvious effect on exposure of PE_PGRSs in the mycobacterial surface [13]. The finding that surface localization of PE_PGRS proteins could be achieved in *M. smegmatis* which lacks ESX5 [20], [46], further questions the need of ESX5 for proper PE_PGRS cellular localization.

In order to investigate PE_PGRS30 localization in different mycobacterial species, we expressed in *M. smegmatis*, *M. bovis* BCG and *Mtb* the PE_PGRS30^GFP^ protein under the control of its own putative promoter [27]. Surprisingly, PE_PGRS30^GFP^ polarized at the bacterial poles when expressed in *Mtb* and *M. bovis* BCG but not in *M. smegmatis*, contrary to what previously observed when PE_PGRS30 was overexpressed under the control of the *hsp60* promoter in *M. smegmatis* [46], [47]. These results highlight the impact that protein expression has on polarization and most importantly, that segregation of PE_PGRS30 at the bacterial poles occurs in members of MTB complex, which naturally express PE_PGRS, but not in *M. smegmatis*, which does not express any PE_PGRS, and as such may miss some of the protein partners [28], [48] or cellular components involved in MTB complex in PE_PGRS cellular localization [9]. It remains to be seen whether polarization of PE_PGRS30 is dependent upon interaction with ESX-5 components and expression of the chimeras used in this study in *Mtb* ΔESX5 mutants [13], [49] will shed light on the molecular mechanism of this process. The fact that a similar pattern of protein localization for PE_PGRS30 and its functional deletion chimeras was observed in *Mtb* and BCG suggests that lack of ESX-1 or other region of deletions in BCG [45], [50] does not impact PE_PGRS30 localization.

PE_PGRS30 polarization was observed also for the _30_PE_PGRSΔCT^GFP^, indicating that the unique 306 amino acids C-terminal domain is not necessary for proper localization of the protein on the mycobacterial cell wall. These results are in line with our previous finding that the C-terminal unique domain is dispensable for the PE_PGRS30-dependent virulence [27] and imply that the PGRS domain is properly exposed or available to deploy its function in *Mtb* regardless of the C-terminal domain. Conversely, deletion of the PGRS central domain, as in the _30_PE^GFP^ and _30_PE_Ct^GFP^ chimeras, results in a partial loss of the polar phenotype, suggesting that both the PE and PGRS domains are important for proper PE_PGRS30 localization. Since the first 140 amino acids of the protein (PE domain) are likely responsible for protein translocation [19]–[21], but not sufficient to mediate polar localization, it implies that the PGRS region downstream of the GRPLI domain plays a key role in protein polarization.

The importance of the PGRS region and of the GRPLI domain is further highlighted by the analysis of the *Mtb* cell lysates expressing the PE_PGRS30^HA^ and its functional HA-tagged chimeras in immunoblot using antiserum directed against the C-terminal domain. A clear band of 52 kDa was detected in all *Mtb* lysates, corresponding probably to the gene product of Rv3812, which was annotated as PE_PGRS62 [15] although it lacks both the typical PGRS domain and the GRPLI anchoring domain, highly conserved in all PE_PGRS proteins [18], [35], [51]. While the signal of the 52 kDa band was very similar in intensity to that corresponding to _30_PE_CT^HA^, a much lower signal was detected at ≈88 kDa, which corresponds to the full length PE_PGRS30. Since the level of fluorescence in *Mtb* was found similar between PE_PGRS30^GFP^ and _30_PE_CT^GFP^ (figure 2), indicating similar level of protein expression, it is possible that PE_PGRS30 remains associated with the non-soluble cellular debris, suggesting a tight anchoring to the mycobacterial cell wall. Conversely, the _30_PE_CT^HA^, similarly to the Rv3812 gene product, appears to be more soluble, further highlighting the importance of PGRS for proper cellular localization.

Indeed, immunofluorescence studies showed that in *Mtb* the _30_PE_CT^HA^ chimera is exposed on the mycobacterial surface while the full length protein (PE_PGRS30^HA^) is not. Hence, while the results obtained with the _30_PE_CT^HA^ support previous findings showing that the PE domain of PE_PGRS33 contains the information sufficient to drive an heterologous antigen to the mycobacterial surface [20], [37], the results obtained with the full length protein suggest that the unique C-terminal domain of PE_PGRS30 localizes in the periplasm or is tightly embedded in the mycomembrane. The lack of any information on the potential function of the 306 amino acids long C-terminal domain prevents further hypothesis and the 40% identity (ClustalW2 software) with the C-terminal domain of Rv3812 is not high enough to exclude a different function.

Under physiological conditions of expression, PE_PGRS30^GFP^ and PE_PGRS33^GFP^ showed a different localization pattern in *Mtb*, with the former strongly polarizing and the latter homogeneously dispersed throughout the bacterial cell. Surprisingly, _33_PE^GFP^ polarized at the cell poles but not _30_PE^GFP^. These results indicate that concentration of PE_PGRS30 at the bacterial poles depends upon the PGRS domain and not on the PE domain. Differences in amino acids sequences (figure S4) between the _30_PE and _33_PE may help explain the differential pattern of polarization observed between _30_PE^GFP^ and _33_PE^GFP^, though we should remind that no such proteins, that is PE domain containing the GRPLI motif but lacking the PGRS domain (corresponding to the first ≈140 amino acids of PE_PGRSs), are naturally expressed by *Mtb* and as such these functional deletion chimeras may be missing key protein domains required to reach the natural cellular localization. Genomic analysis of MTB complex and *M. marinum* indicate that PE_PGRS30 is much more conserved than PE_PGRS33, with orthologous proteins found in *M. canettii* and *M. marinum*. Conversely, the gene encoding PE_PGRS33 was found only in the MTB complex genome but not in the smooth tubercle bacilli genome [52] nor in *M. marinum* genome, despite the large number of PE_PGRS genes present in the latter [18]. This evolutionary context may help explain the different cellular localization observed in this study between the two PE_PGRS proteins.

Concentration at one bacterial pole of proteins and enzymes involved in peptidoglycan synthesis [53] and virulence, such as the ESX1 secretory apparatus [54], [55] is known to be important in mycobacteria. Concentration at a pole of virulence associated proteins or protein scaffolds may be a key step to evade from phagosome or eject from host cells [56]. Indeed, the ESX1 T7SS apparatus was shown to accumulate at the bacterial pole [54], [55] and this process may be instrumental to produce holes in the phagosome that warrant cytoplasm access to *Mtb* [57]. Our finding indicating that PE_PGRS30 strongly accumulate at the bacterial poles in *Mtb* infecting macrophages and replicating intracellularly suggests that polarization may be a key step in the PE_PGRS30-dependent virulence mechanism. Since PE_PGRS30 is required for the survival and replication of *Mtb* in macrophages [27], it may be hypothesized that PE_PGRS30, alone or in combination with other yet undefined effectors, concentrates at one bacterial pole to maximize its activity. Conversely, PE_PGRS33^GFP^ was homogeneously distributed throughout the bacterial cells during *Mtb* infection in macrophages, indicating that different PE_PGRS proteins show a different localization pattern. These findings further support the view that the PE_PGRS family includes a heterogeneous, differentially regulated group of proteins which, despite their similarities, exert different roles and functions in *Mtb* biology [58]. The repetitive GGA-GGN repeats of the PGRS domain are intercalated by protein-specific sequences which provide each PE_PGRS with a specific role and function. The results of this study highlight the role of the PGRS domain in the cellular localization of an *Mtb* virulence factor as PE_PGRS30.

## Supporting Information

Figure S1 **Immunoblots showing expression of PE_PGRS30 chimeras tagged with green fluorescent protein (GFP) in** ***Mycobacterium smegmatis*** **(A) and in** ***Mycobacterium tuberculosis*** **(B).** Immunoblot analysis of whole cell lysates were probed with anti-GFP primary antibody.
(TIF)

Figure S2 **Polar localization of PE_PGRS30^GFP^ spatial distribution in** ***M. bovis*** **BCG.** A) Confocal images of *M. bovis* BCG expressing PE_PGRS30^GFP^ and its functional GFP-tagged chimeras obtained with a 63× objective. In the inbox, a 100× image obtained overlapping green channel and transmission image is shown. B) Sample line profile obtained quantifying the fluorescence along mycobacterial cell. C) Ratio between the GFP emission intensity at bacterium pole (considered as the value 200 nm far from the bacterium border) and GFP emission intensity in the cytoplasm (measured at the 50% of the bacterium length). Twenty R values were analyzed for each *M. bovis* strain under study. Two-tailed Student's *t*-test was used to analyze R ratio (* *p*<0.05, ** *p<*0.01).
(TIF)

Figure S3 A) Schematic representation showing the DNA construct ptPA^30Cter^ used to immunize mice and obtain specific polyclonal serum against the unique C-terminal domain of PE_PGRS30. B) Schematic showing the protein domains of PE_PGRS30 and PE_PGRS62.
(TIF)

Figure S4 **Alignment of amino acid sequence of _30_PE and _33_PE using** ***ClustalW2 software*** **and** ***ESPripte software*** **.**
(TIF)

Table S1 **Primers used in this work.**
(TIF)

Table S2 **R indicates the index of the polarization of the protein distribution calculated with the ratio I_pole_/I_cyto_ where I_pole is the GFP emission intensity at bacterium pole, considered as the value in correspondence of the border of the bacterium, and I_cyto_ is the GFP emission intensity in the middle part of the bacterium (measured at the 50% of the bacterium length).**
(TIF)
