# Supplementary figures and images for: Impact of Protein Domains on PE_PGRS30 Polar Localization in Mycobacteria
Source: PLoS One. 2014 Nov 12;9(11):e112482. doi: 10.1371/journal.pone.0112482 (PMC4229189; doi:10.1371/journal.pone.0112482)

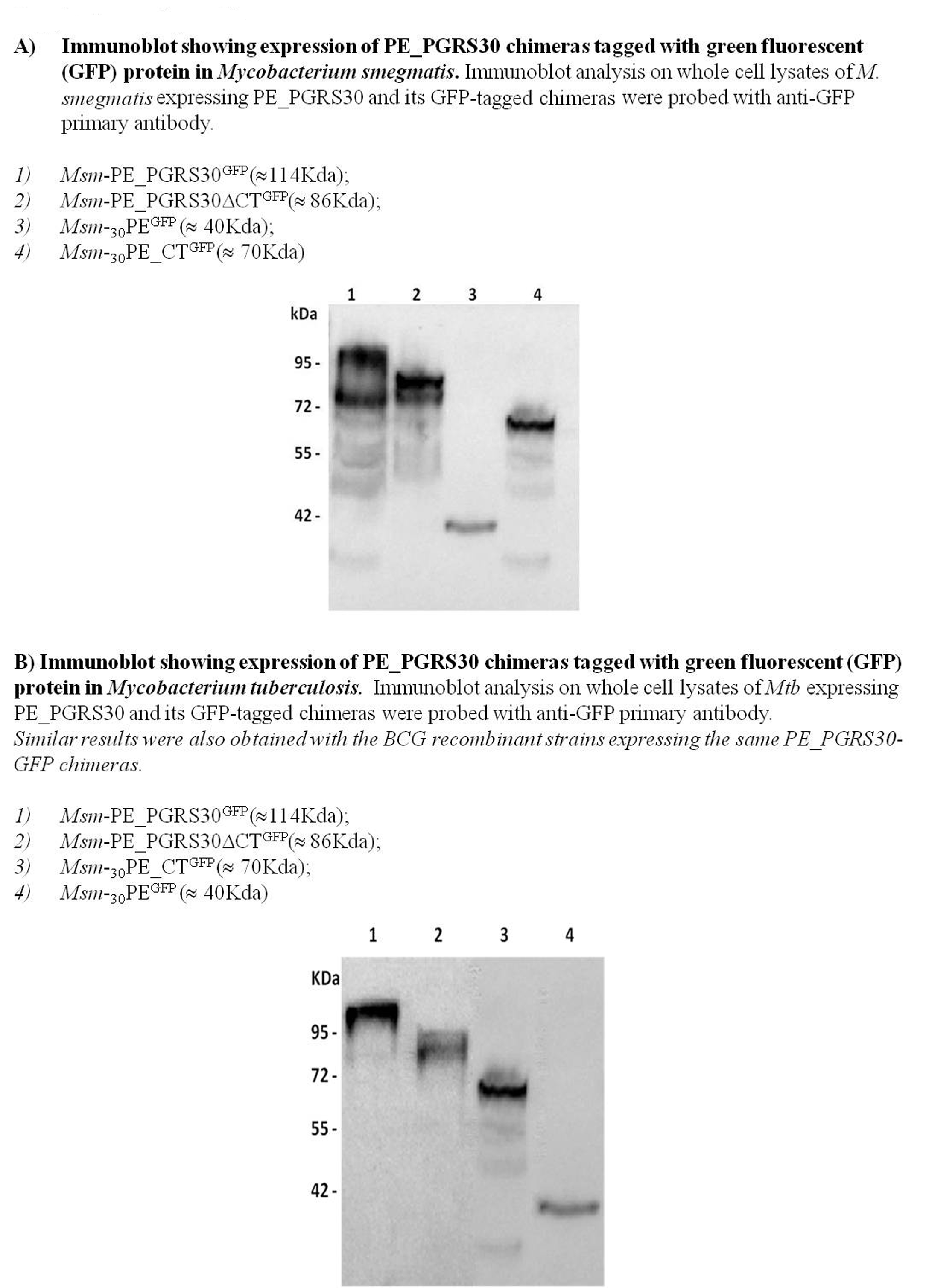

Supplement: Figure S1 — Immunoblots showing expression of PE_PGRS30 chimeras tagged with green fluorescent protein (GFP) in Mycobacterium smegmatis (A) and in Mycobacterium tuberculosis (B). Immunoblot analysis of whole cell lysates were probed with anti-GFP primary antibody. (TIF) [file pone.0112482.s001.tif]

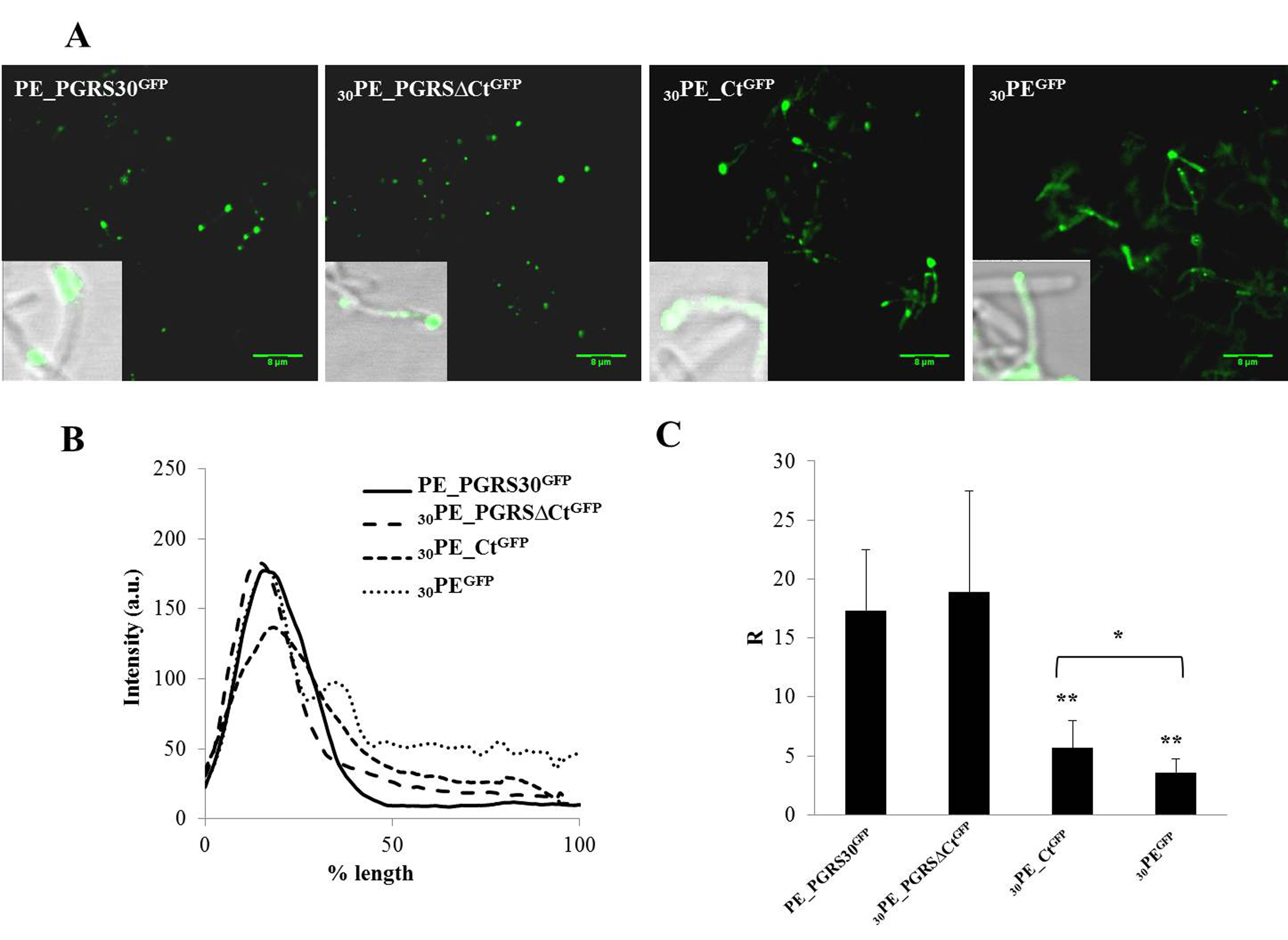

Supplement: Figure S2 — Polar localization of PE_PGRS30GFP spatial distribution in M. bovis BCG. A) Confocal images of M. bovis BCG expressing PE_PGRS30GFP and its functional GFP-tagged chimeras obtained with a 63× objective. In the inbox, a 100× image obtained overlapping green channel and transmission image is shown. B) Sample line profile obtained quantifying the fluorescence along mycobacterial cell. C) Ratio between the GFP emission intensity at bacterium pole (considered as the value 200 nm far from the bacterium border) and GFP emission intensity in the cytoplasm (measured at the 50% of the bacterium length). Twenty R values were analyzed for each M. bovis strain under study. Two-tailed Student's t-test was used to analyze R ratio (* p<0.05, ** p<0.01). (TIF) [file pone.0112482.s002.tif]

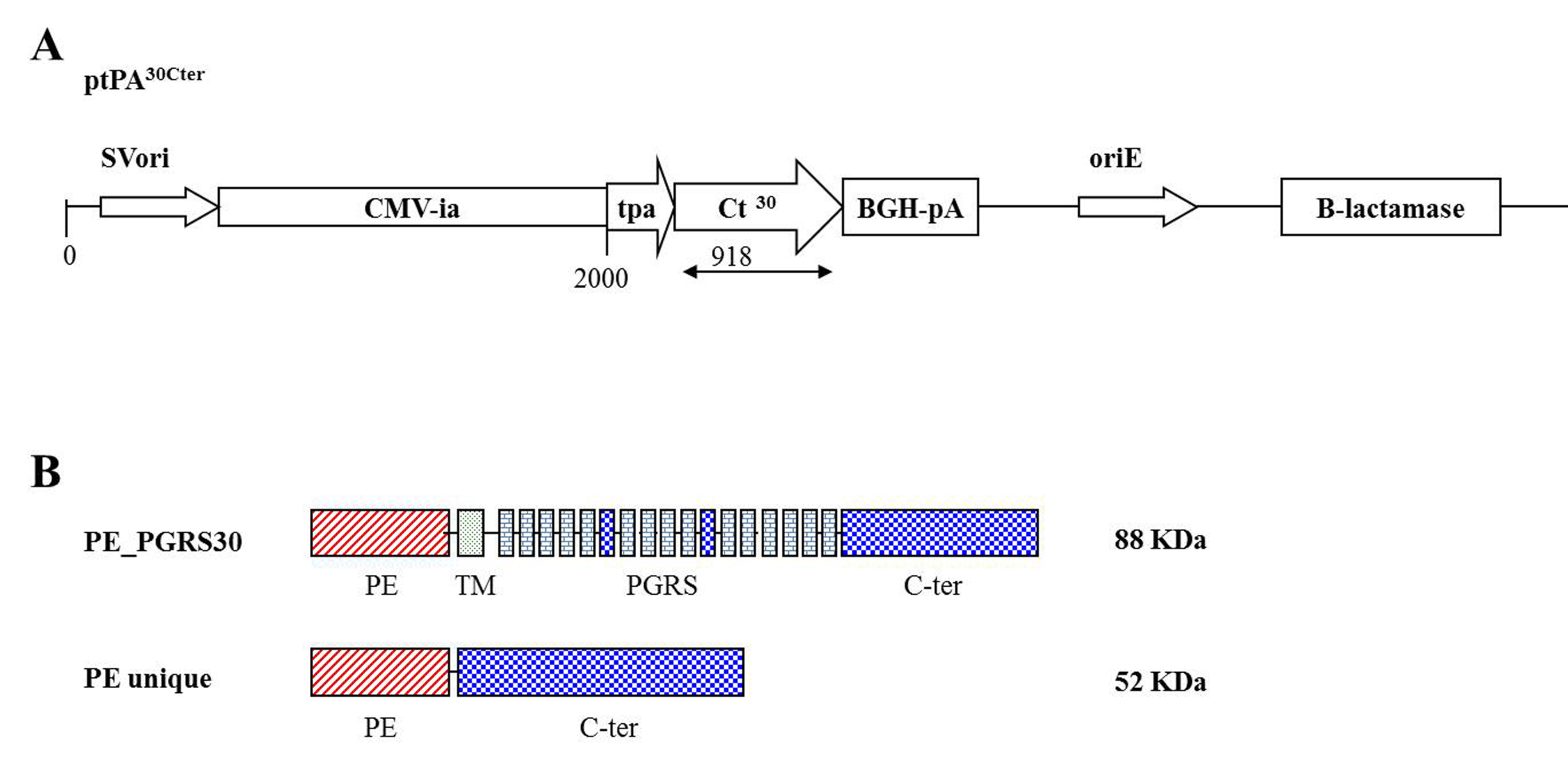

Supplement: Figure S3 — A) Schematic representation showing the DNA construct ptPA30Cter used to immunize mice and obtain specific polyclonal serum against the unique C-terminal domain of PE_PGRS30. B) Schematic showing the protein domains of PE_PGRS30 and PE_PGRS62. (TIF) [file pone.0112482.s003.tif]

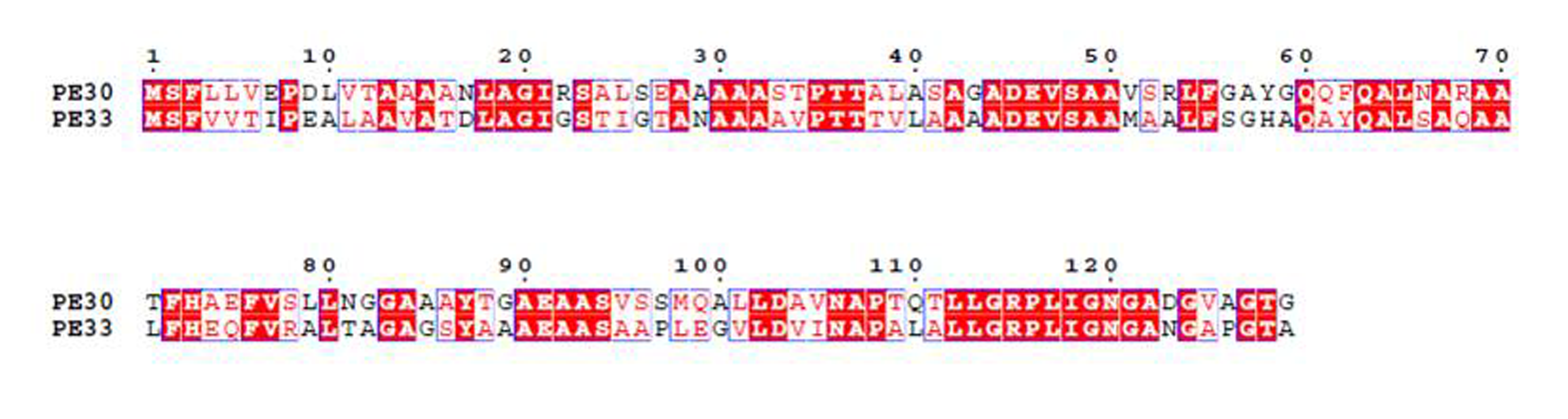

Supplement: Figure S4 — Alignment of amino acid sequence of 30PE and 33PE using ClustalW2 software and ESPripte software . (TIF) [file pone.0112482.s004.tif]

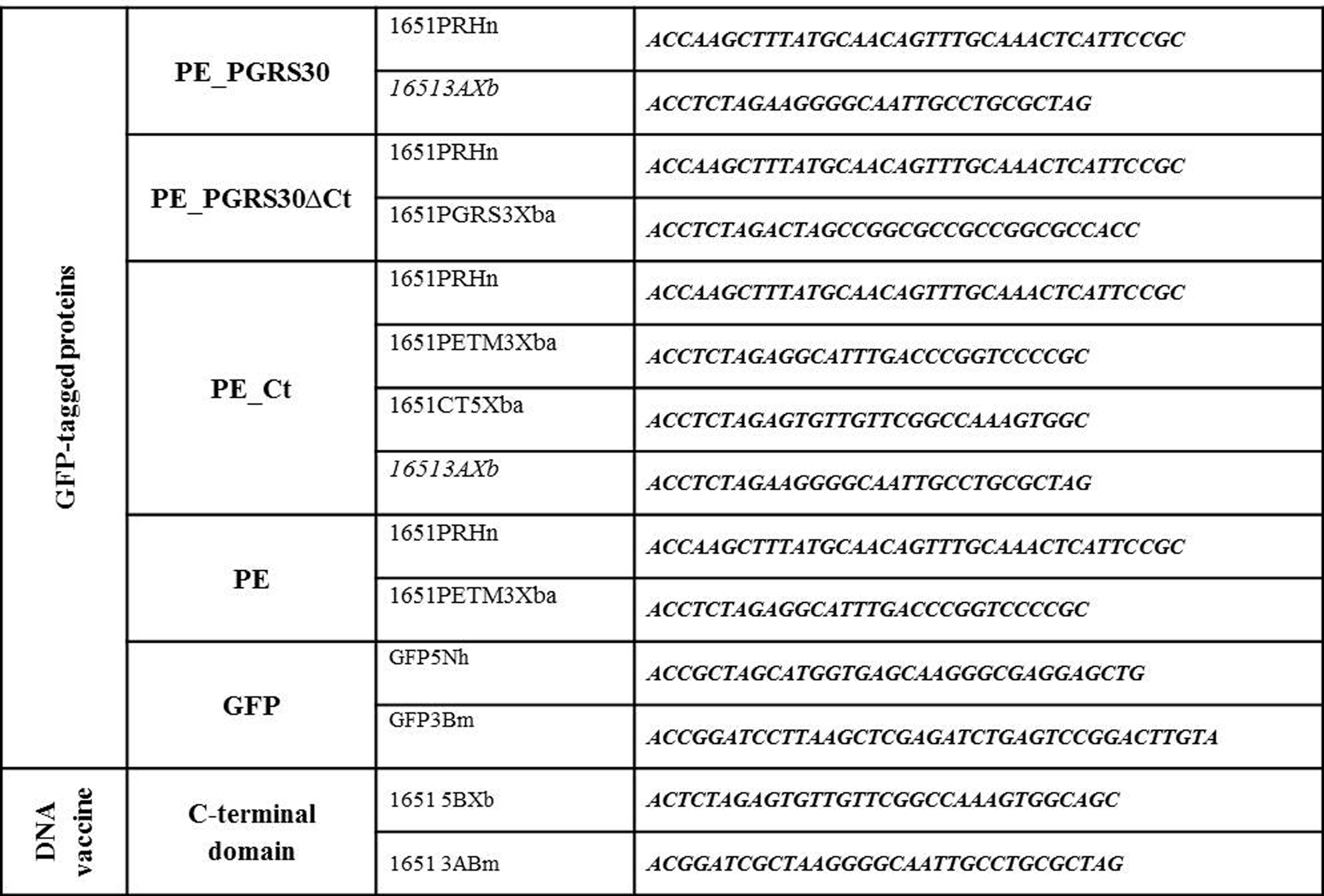

Supplement: Table S1 — Primers used in this work. (TIF) [file pone.0112482.s005.tif]

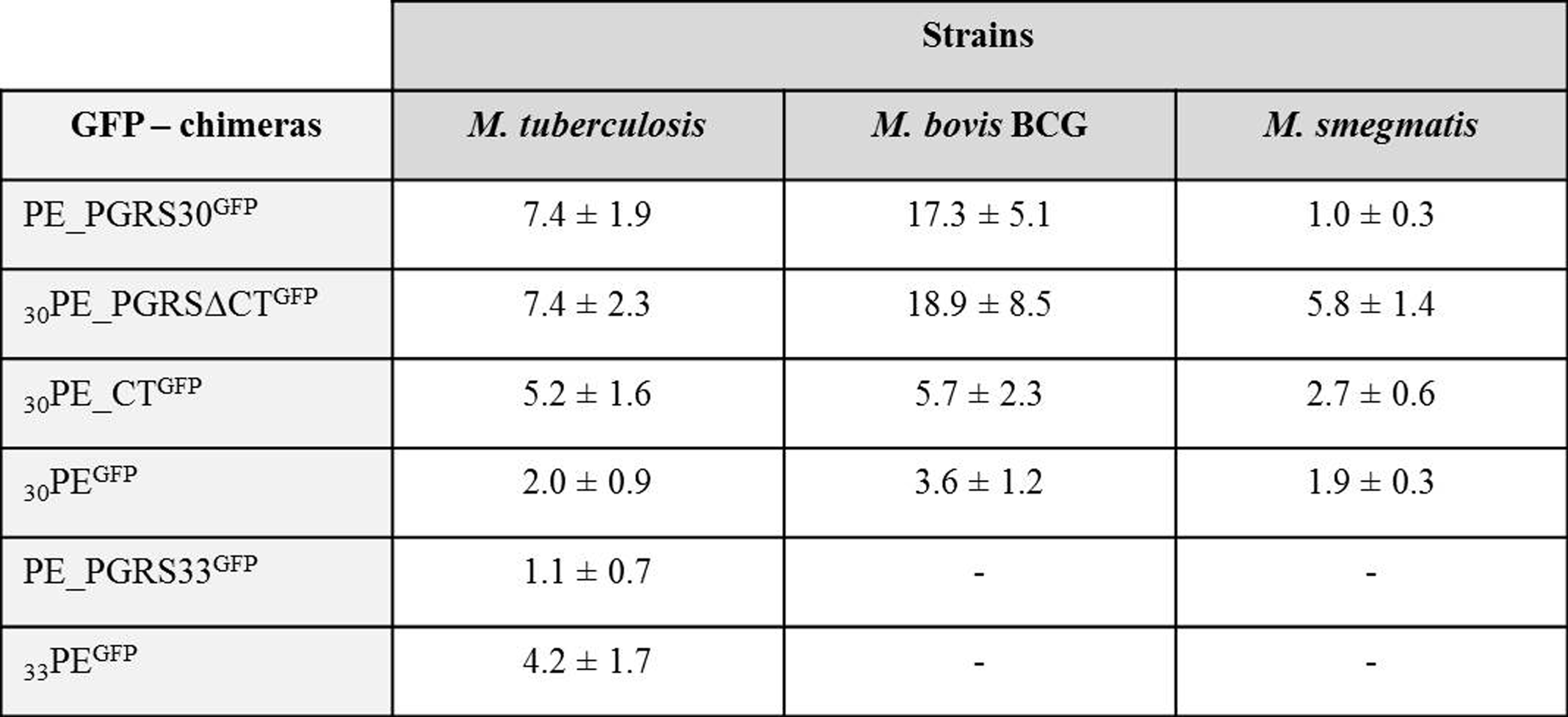

Supplement: Table S2 — R indicates the index of the polarization of the protein distribution calculated with the ratio Ipole/Icyto where I_pole is the GFP emission intensity at bacterium pole, considered as the value in correspondence of the border of the bacterium, and Icyto is the GFP emission intensity in the middle part of the bacterium (measured at the 50% of the bacterium length). (TIF) [file pone.0112482.s006.tif]
